# Prediction of a time-sensitive condition among patients with dizziness assessed by the emergency medical services

**DOI:** 10.1186/s12873-021-00423-5

**Published:** 2021-03-25

**Authors:** C. Magnusson, J. Gärskog, E. Lökholm, J. Stenström, R. Wetter, C. Axelsson, M. Andersson Hagiwara, N. Packendorff, K. Jood, T. Karlsson, J. Herlitz

**Affiliations:** 1 Department of Molecular and Clinical Medicine, Institute of Medicine, Sahlgrenska Academy, University of Gothenburg, Gothenburg, Sweden; 2 Department of Prehospital Emergency Care, Sahlgrenska University Hospital, Gothenburg, Sweden; 3 Centre for Prehospital Research, Faculty of Caring Science, Work Life and Social Welfare, University of Borås, Borås, Sweden; 4 Department of Clinical Neuroscience and Physiology, Sahlgrenska Academy, University of Gothenburg, Gothenburg, Sweden; 5 Health Metrics Unit, Institute of Medicine, Sahlgrenska Academy, University of Gothenburg, Gothenburg, Sweden

**Keywords:** Prehospital, Dizziness, Outcome, Diagnosis, Time sensitive

## Abstract

**Background:**

Dizziness is a relatively common symptom among patients who call for the emergency medical services (EMS).

**Aim:**

To identify factors of importance for the early identification of a time-sensitive condition behind the symptom of dizziness among patients assessed by the EMS.

**Methods:**

All patients assessed by the EMS and triaged using Rapid Emergency Triage and Treatment (RETTS) for adults code 11 (=dizziness) in the 660,000 inhabitants in the Municipality of Gothenburg, Sweden, in 2016, were considered for inclusion. The patients were divided into two groups according to the final diagnosis (a time-sensitive condition, yes or no).

**Results:**

There were 1536 patients who fulfilled the inclusion criteria, of which 96 (6.2%) had a time-sensitive condition. The majority of these had a stroke/transitory ischaemic attack (TIA). Eight predictors of a time-sensitive condition were identified. Three were associated with a reduced risk: 1) the dizziness was of a rotatory type, 2) the dizziness had a sudden onset and 3) increasing body temperature. Five were associated with an increased risk: 1) sudden onset of headache, 2) a history of head trauma, 3) symptoms of nausea or vomiting, 4) on treatment with anticoagulants and 5) increasing systolic blood pressure.

**Conclusion:**

Among 1536 patients who were triaged by the EMS for dizziness, 6.2% had a time-sensitive condition. On the arrival of the EMS, eight factors were associated with the risk of having a time-sensitive condition. All these factors were linked to the type of symptoms or to clinical findings on the arrival of the EMS or to the recent clinical history.

**Supplementary Information:**

The online version contains supplementary material available at 10.1186/s12873-021-00423-5.

## Background

Dizziness is a relatively common symptom among patients who seek emergency care. Many of these patients dial 112, which is the telephone number when ambulance transport to hospital is required in Sweden. Among patients assessed by the EMS, it is important to determine whether they have a time-sensitive condition, i.e. a condition that requires immediate medical intervention either in the emergency department or, for example, in the cathlab.

Dizziness is a unifying concept for a number of different experiences for the patient, such as being on a carousel, off balance, near syncope or motion of the sea. It has previously been reported that about 3 % of patients who visit the emergency department do so because of dizziness [1]. We recently reported that about 3 % of patients seen by the EMS are assessed as suffering from dizziness [2].

Dizziness may arise from a number of causes including a local aetiology, as well as systemic factors. Some of the underlying aetiologies may require immediate medical intervention after arriving in hospital, i.e. time-sensitive conditions.

Damage to central or peripheral parts of the vestibular nerve will generate an acute vestibular syndrome, which consists of several symptoms, such as dizziness, nausea/vomiting, nystagmus and trouble maintaining body balance.

When the damage is localised to the inner ear or in the vestibular nerve, there is a peripheral aetiology. Examples of a peripheral aetiology of an acute vestibular syndrome are benign paroxysmal positional vestibular neuritis, Meniere’s disease, bacterial labyrinthitis and herpes zoster oticus [3].

Dizziness of central origin is localised in the central parts of the vestibular system in the brain stem and/or cerebellum and the underlying aetiologies include TIA/stroke, migraine, tumour in the brain stem, encephalitis and multiple sclerosis.

It has in fact been shown that, among patients with TIA/stroke, when the posterior circulation is affected, they relatively often present with dizziness. These patients have significant mortality and morbidity.

However, it has been suggested that the majority of patients with acute dizziness have aetiologies other than damage to the peripheral or central parts of the vestibular system. In one large study, it was reported that 63% of all cases with acute dizziness had other aetiologies. The most common aetiology was an upper airway infection (35%) and the second most common was hypertension (18%). Time-sensitive conditions, such as bradycardia, AV block III, sepsis and acute coronary syndrome, accounted for 3 % of all cases [4]. Similar findings have been made by others reporting that more than half the patients with dizziness have an aetiology which is not related to the vestibular system, with the majority having an internal medicine disorder [5].

Time-sensitive conditions which are not related to the vestibular system may still cause dizziness; they include TIA/stroke, water-electrolyte imbalance, arrhythmia, carbon monoxide poisoning and aortic dissection.

The variety of conditions that may exist behind symptoms of dizziness highlight the difficulties healthcare providers experience when attempting to differentiate these symptoms into benign and malignant conditions when they meet patients with these symptoms.

The burden on the emergency medical services (EMS) has increased markedly during the last few decades. This is primarily explained by an elderly population with an often extensive comorbidity and the fact that people nowadays tend to dial 112 more often than before, despite not suffering from a time-sensitive condition [6]. It has also been shown that not all these patients require transport to hospital [7] and that some could preferably be handled by a lower level of care [7].

This situation constitutes a demanding challenge for the EMS staff who, to ensure the adequate utilisation of resources, must try to differentiate time-sensitive from non-time-sensitive conditions already at the scene.

Patients with dizziness constitute a cohort in which the EMS clinician may have difficulty making an appropriate assessment. Although a large proportion of these patients appear to suffer from a relatively benign condition, there are time-sensitive conditions hidden among them which are important to identify.

The aim of this study was, among patients who were assessed by the EMS and triaged using Rapid Emergency Triage and Treatment (RETTS) for adults and given the Emergency Signs and Symptoms (ESS) code of dizziness, to attempt to identify clinical factors that may help to differentiate patients who are suffering from a time-sensitive condition from those that are not.

## Methods

### Design

This study is a retrospective, observational study in which patients with on-the-scene Pre-hospital Emergency Nurse (PEN) triage of RETTS code no 11 (dizziness) were included for a manual review.

### Recruitment of patients

All the patients who were seen by the EMS in the Municipality of Gothenburg and given the rapid emergency triage and treatment system for adults (RETTS-A) code 11 = dizziness (*n* = 2048) from 1 January to 31 December 2016 were included in the study.

**Inclusion criteria**

1. Primary mission and assessed by a PEN
2. Given RETTS-A code 11 by a PEN

**Exclusion criteria**

1. Age < 16 years
2. Patient primarily assessed by another caregiver, for example, a physician or at an outpatient clinic
3. Not assessed by a physician at the hospital
4. Incomplete identification number
5. Patient sent to another hospital outside the catchment area

### The EMS system in Sweden

The health care provided in Sweden, including pre-hospital care, is tax funded. The EMS organisation uses national/regional guidelines. Each ambulance in Sweden should be staffed by at least one registered nurse (RN), responsible for the care. The RN often has an additional one-year postgraduate education in pre-hospital emergency care (PEN). However, ambulance crew set-ups can take the form of two nurses or one nurse and one emergency medical technician (EMT). The PEN assesses the patient at the scene and has approximately 40 different types of drug at his/her disposal. The PEN is responsible for deciding on the level of care, which means that not all patients are transported to hospital [8].

Ambulances are dispatched from a dispatch centre with three priorities as previously described [9].

### Triage system

The RETTS-A [10] is a five-level triage system currently in use in the majority of emergency departments (EDs) and EMS organisations in Sweden. However, it is not used by the dispatch centre but only after the arrival of the EMS at the scene. The RETTS-A is developed, licensed and maintained by a Swedish company (Predicare AB). The RETTS-A contains 58 charts with common ED presentations. Each chart includes both emergency signs and symptoms (ESS) and vital signs (VS), as previously described (7). The level of severity of both VS and ESS is divided into the colours of red, orange, yellow, green and blue, but blue is not used by the EMS. Triage level red is considered life threatening, resulting in immediate contact with a physician, orange is potentially life threatening, while yellow and green can wait in the ED without medical risk. Yellow is considered to be more urgent than green. The highest triage level of either VS or ESS becomes the final triage level.

### Type of vertigo

The type of vertigo was divided into four categories according to the free text based on the ED physician’s assessment.

1. Rotatory dizziness = the sensation of being on a merry-go-round
2. Balance disturbance = the sensation of unsteadiness or sensation of falling
3. Nautical dizziness = the sensation that the ground tilts up and down, as when travelling in a boat
4. Non-specified dizziness = vague presentation, lightheadedness or unspecific dizziness including the remainder of patients who did not have any of the other three sensations described

### Data collection

Data were collected from digital case records in the EMS data system and the hospital medical records. The PENs complete the case records in two steps. During the mission, they make notes in a paper-based system and, after handover at the ED, they complete the digital case record on a desktop located in the ED.

The final diagnosis at discharge was collected from the hospital medical records. Patients were then divided into two groups, according to whether or not they had a time-sensitive condition. A time-sensitive condition was defined if the final diagnosis was any of those described by Wibring et al. [11]. TIA/stroke was of particular interest for this article.

### Data analyses

Univariate comparisons between patients with and patients without a time-sensitive condition were performed using the Mann-Whitney U test for continuous/ordered variables and Fisher’s exact test for dichotomous variables.

With the exception of the two variables of “non-transported, attended the ED within 72 hours” and “EMS on-the-scene time”, which were regarded as outcomes rather than predictors, all variables with a univariate *p* <  0.20 for differences between the two groups were tested for inclusion in a multiple logistic regression model, using backward stepwise selection with *p* <  0.01 for staying in the model. This procedure was performed both using only complete cases and, due to the amount of missing data for several of the variables, using multiple imputations (primary analysis). For the latter, which was regarded as the primary analysis, missing data were assumed to be missing at random (MAR) and 50 imputed datasets were generated with the Markov Chain Monte Carlo (MCMC) method using the expectation-maximisation (EM) algorithm. Rubin’s rules were used to pool the results from the imputed datasets. To identify independent predictors of time-sensitive conditions in the multiple imputation multivariable analysis, we started with a model including all the variables in Table 1 with a univariate *p* <  0.20. Collinearity was checked by association measurements between variables, as well as by inspecting the variance inflation factor, condition index and eigenvector proportions in a multiple linear regression model including all the candidate variables. One important collinearity, between systolic and diastolic blood pressure, was found and, as a result, diastolic blood pressure was excluded from the following analysis. Multiple logistic regression was performed in each of the 50 imputed datasets and the variable with the highest *p*-value in the pooled result was excluded from the model. A new regression analysis was then performed in each imputed dataset and, of the remaining variables, the one with the highest *p*-value in the pooled result was excluded. This procedure was repeated until all the remaining variables yielded a p-value below 0.01 in the pooled result.

**Table 1:** Patient characteristic and EMS assessment with and without a time-sensitive condition not included in the multivariable analysis

	Total	Not time-sensitive condition	Time-sensitive condition	*P*
	*n* = 1536	*n* = 1440	*n* = 96	
**Non-transported patients – n (%)**				
Attended the ED within 72 h	43 (12.3)^1^	38 (2.6)	5 (5.2)	0.186
**Mode of transport – n (%) (38,5)**^**2**^				0.285
Ambulance	1361 (91.2)	1277 (91.1)	84 (92.3)	
Patient transport vehicle	50 (3.3)	48 (3.4)	2 (2.2)	
Seated transport vehicle	49 (3.3)	47 (3.4)	2 (2.2)	
Single responder	17 (1.1)	15 (1.1)	2 (2.2)	
By own means	16 (1.1)	15 (1.1)	1 (1.1)	
**EMS time – median h:mm (176,13)**				
Dispatch – arrival in hospital	0:53 (0:44,1:05)	0:54 (0:44,1:05)	0:53 (0:45,1:07)	0.481
Time on scene	0:22 (0:16,0:29)	0:22 (0:16,0:29)	0:25 (0:18,0:31)	0.006
**Vital signs – median (10th, 90th percentile)**				
Respiratory rate/min (73,4)^2^	17 (14,20)	17 (14,20)	16 (14,20)	0.539
Heart rate/min (47,5)	80 (62,104)	80 (62,104)	78 (60,100)	0.408
**Medical history – n (%)**^**3**^ **(19,0)**				
Atrial fibrillation	241 (15.9)	223 (15.7)	18 (18.8)	0.470
Myocardial infarction	159 (10.5)	147 (10.3)	12 (12.5)	0.491
Angina pectoris	98 (6.5)	92 (6.5)	6 (6.3)	1.000
Heart failure	73 (4.8)	69 (4.9)	4 (4.2)	1.000
Peripheral vascular disease	23 (1.5)	22 (1.5)	1 (1.0)	1.000
Cancer	152 (10.0)	142 (10.0)	10 (10.4)	0.861
**History of presenting complaint – n (%)**^**4**^ **(19,0)**				
Transient loss of consciousness	134 (8.8)	124 (8.7)	10 (10.4)	0.576
Recurrent transient loss of consciousness	31 (2.0)	29 (2.0)	2 (2.1)	1.000
Headache	357 (23.5)	333 (23.4)	24 (25.0)	0.710

ED: emergency department

^1^Denoted as percentage of non-transported patients (*n* = 351)

^2^Missing data for the groups of not time-sensitive conditions and time-sensitive conditions respectively

^3^A patient could have more than one medical history diagnosis

^4^A patient could have more than one symptom

Two-sided tests were used and *p*-values below 0.01 were considered statistically significant. All univariable analyses were performed using SPSS version 25 and, for the multivariable analyses, SAS for Windows version 9.4 was used.

### Ethical issues

This study was approved by the Regional Ethical Review Authority in Gothenburg, approval no. 970–15. For the retrospective analysis of this register study, informed consent was waived. However, at the time of EMS assessment, patients who asked for their data to remain confidential were not included in the retrospective analysis.

## Results

**Fig. 1:**
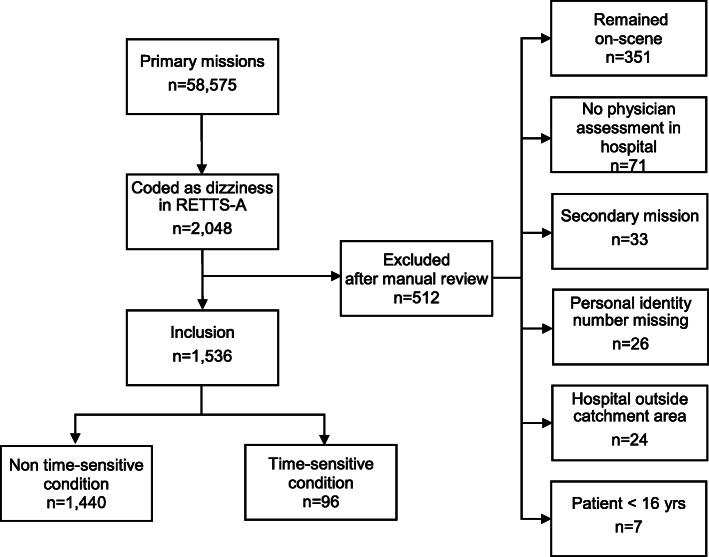
Flow scheme for the study cohort

Overall, there were 59,000 primary missions for the EMS in 2016. Of them, 2048 (3.5%) were coded as dizziness according to the RETTS-A (code 11) (Fig. 1).

After applying inclusion and exclusion criteria, 1536 patients remained. The clinical characteristics of the included patients are shown in Table 1. The overall median age was 73 years and 58% were women.

Of the 1536 included patients, 96 (6.2%) fulfilled the criteria for a time-sensitive condition (Fig. 1). The most frequent time-sensitive conditions were stroke and TIA (*n* = 84), followed by AV block (*n* = 4), electrolyte imbalance (n = 4), a traumatic brain bleed (*n* = 3) and acute coronary syndrome (*n* = 1).

### Univariable analyses

The results of univariable analyses of the association between clinical variables and time-sensitive conditions are shown in Tables 1 and 2.

**Table 2:** Patient characteristics and EMS assessment of patients with and without a time-sensitive condition included in the multivariable prediction model

	Total	Not time-sensitive condition	Time-sensitive condition	*P*
	n = 1536	n = 1440	n = 96	
**Age – years (25th, 75th percentile) (12,0)**^**1**^				
Median	73 (57,83)	73 (57,83)	78 (66,85)	0.007
**Gender – n (%) (13,0)**				
Female	877 (57.6)	830 (58.2)	47 (49.0)	0.088
**Dispatcher priority – n (%) (1,0)**				0.038
Priority 1	523 (34.0)	481 (33.4)	42 (43.8)	
Priority 2	945 (61.6)	894 (62.1)	51 (53.1)	
Priority 3	67 (4.4)	64 (4.5)	3 (3.1)	
**Triage level according to RETTS-A – n (%)**				0.026
Red	22 (1.4)	22 (1.5)	0 (0.0)	
Orange	415 (27.0)	375 (26.0)	40 (41.7)	
Yellow	878 (57.2)	835 (58.0)	43 (44.8)	
Green	221 (14.4)	208 (14.5)	13 (13.5)	
**Vital signs – median (10th 90th percentile)**				
Oxygen saturation, % (49,5)^1^	98 (95,100)	98 (95,100)	98 (95,99)	0.063
Systolic blood pressure, mm/hg (51,4)	150 (110,190)	145 (110,187)	160 (130,190)	< 0.001
Diastolic blood pressure, mm/hg (142,8)	80 (70,100)	80 (70,100)	90 (70,104)	0.026
Body temperature °C (83,7)	36.7 (36.0,37.3)	36.7 (36.0,37.3)	36.6 (35.6,37.2)	0.189
**Blood glucose recorded – n (%)**	739 (48.1)	685 (47.6)	54 (56.3)	
Elevated blood glucose > 9.4 mmol/l	117 (15.8)	104 (15.2)	13 (24.1)	0.118
**Medical history – n (%)**^**2**^ **(19,0)**				
Stroke	189 (12.5)	169 (11.9)	20 (20.8)	0.016
Transient ischaemic attack	95 (6.3)	85 (6.0)	10 (10.4)	0.122
Hypertension	618 (40.7)	568 (40.0)	50 (52.1)	0.024
Diabetes	213 (14.0)	195 (13.7)	18 (18.8)	0.172
**History of presenting complaint – n (%)**^**3**^ **(19,0)**				
Sudden onset^4^	1165 (76.8)	1104 (77.7)	61 (63.5)	0.003
Nausea, vomiting	801 (52.8)	742 (52.2)	59 (61.5)	0.091
Sudden onset headache^4^	35 (2.3)	24 (1.7)	11 (11.5)	< 0.001
Head trauma	86 (5.7)	70 (4.9)	16 (16.7)	< 0.001
Treatment with anticoagulants	215 (14.2)	191 (13.4)	24 (25.0)	0.004
**Types of dizziness – n (%) (402,16)**				< 0.001
Rotatory vertigo	445 (39.8)	428 (41.2)	17 (21.2)	
Balance disturbance	85 (7.6)	81 (7.8)	4 (5.0)	
Nautical dizziness	111 (9.9)	102 (9.8)	9 (11.3)	
Non-specific dizziness	477 (42.7)	427 (41.2)	50 (62.5)	

^1^Missing data for the groups of not time-sensitive conditions and time-sensitive conditions respectively

^2^A patient could have more than one medical history diagnosis

^3^A patient could have more than one symptom

^4^Onset within a few hours

Patients with a time-sensitive condition were significantly older. They also had higher systolic blood pressure, more often had ongoing anticoagulation, more often described a sudden onset of headache but less often described a sudden onset of dizziness and less often had dizziness of the rotatory type, nautical, or balance disturbing. Moreover, they more frequently had a history of head trauma.

### Multivariable analyses (Table 3)

There were eight factors that were independently associated with the risk of a time-sensitive condition. Two factors, the rotatory type of dizziness and the sudden onset of symptoms, were both associated with a threefold decrease in the risk of a time-sensitive condition. Furthermore, the risk of a time-sensitive condition was reduced by nearly 50% for each degree(C) of increase in body temperature.

**Table 3:** Multivariable analysis (backward stepwise selection, *p* < 0.01 for staying in model)

	Multiple imputations (n = 96 + 1440)	
	OR (95% CI)	*P*
Systolic blood pressure (per mmHg)	1.015 (1.007,1.022)	0.0001
Body temperature (per degree Celsius)	0.56 (0.38,0.82)	0.003
Rotary vertigo	0.32 (0.18,6.59)	0.0002
Sudden onset	0.35 (0.21,0.57)	< 0.0001
Nausea, vomiting	2.10 (1.29,3.43)	0.003
Sudden onset headache	8.54 (3.71,19.67)	< 0.0001
History of head trauma	4.13 (2.17,7.86)	< 0.0001
Treatment with anticoagulants	2.36 (1.39,3.99)	0.001

OR: odds ratio; CI: confidence interval

The following five factors were associated with an increased risk of a time-sensitive condition: 1) sudden onset of headache with a ninefold increase in risk, 2) a history of head trauma with a fourfold increase in risk, 3) symptoms of nausea or vomiting with a twofold increase in risk, 4) on treatment with anticoagulants with a twofold increase in risk and 5) systolic blood pressure with a 1.5% increase in risk per mmHg increase in systolic blood pressure.

Independent risk factors for a time-sensitive condition were also assessed in the subset of patients with a non-specific dizziness. Results remained unchanged in principle and no additional risk factor appeared (Supplementary Table 2).

## Discussion

We found that, among 59,000 primary missions for the EMS in the catchment area, 3.5% were reported as suffering from dizziness according to the given ESS code. Of them, about 6 % fulfilled the criteria for having a time-sensitive condition, among which the majority had TIA/stroke. On the arrival of the EMS, there were eight factors that were associated with the risk of having a time-sensitive condition. Three factors, i.e. having a rotatory type of dizziness, having a sudden onset of symptoms and increasing body temperature, were all associated with a decreased risk of a time-sensitive condition.

Five factors, i.e. sudden onset of headache, a history of head trauma, symptoms of nausea or vomiting, on treatment with anticoagulants and increasing blood pressure, were all associated with an increased risk of a time-sensitive condition.

Our finding that around 3 % of patients who were seen by the EMS had symptoms of dizziness is in agreement with previous research which states that about 3 % of patients who visit the emergency department have symptoms of dizziness [1]. Furthermore, Hjälte et al. found that 3 % of patients who called for the EMS did so because of dizziness [12].

The observation that around 6 % of the patients with dizziness had a time-sensitive condition is also within the range that has previously been reported [13–16].

The observation that rotatory dizziness is associated with a reduced risk of a time-sensitive condition is supported to some extent by previous research [14, 17].

Moreover, the observation that the sudden onset of dizziness is associated with a reduced risk of a time-sensitive condition has been reported [18]. However, the proportion of patients with a time-sensitive condition among those with a sudden onset of dizziness has been reported with a frequency varying from 0.7 to 11% [15, 19–21]. In some of the cases, there may have been difficulty deciding how to describe the type of onset of dizziness.

The finding that the risk of a time-sensitive condition decreased with increasing body temperature is difficult to explain. One possible explanation is that, among patients with fever and vertigo, the risk of an underlying infection (not defined as a time-sensitive condition) is more marked.

Sudden onset of headache was strongly associated with an increased risk of a time-sensitive condition. Similar findings were made by Kerber et al. [18]. Others [15, 22, 23] did not report results that supported this statement.

Another factor that increased the risk of a time-sensitive condition was a history of head trauma. Dizziness is a common symptom following head trauma. The dizziness often resolves within weeks, but, in some cases, dizziness can affect the patient for a much longer time [24, 25].

A third factor was the presence of nausea or vomiting. These symptoms are often linked to the symptom of dizziness. The fact that these symptoms are associated with an increased risk of a time-sensitive condition has previously been suggested [18, 26]. There is no clear explanation of why nausea or vomiting should be a risk factor for a more alarming aetiology. Among patients with other symptoms such as chest pain, the presence of nausea or vomiting has been associated with an increased risk of an underlying acute coronary syndrome [26].

A fourth factor that increased the risk of a time-sensitive condition was whether the patient was on chronic treatment with anticoagulants. Although this was not reported by other researchers [15, 23] there is a potential explanation for this finding. It may indicate that the patient has previously suffered from a thromboembolic event or suffers from a disease that is associated with an increased risk of such an event, for example, atrial fibrillation. Somewhat surprisingly, a history of atrial fibrillation did not appear as a risk factor for a time-sensitive condition in our survey.

The last risk factor was increasing systolic blood pressure on the arrival of the EMS. This finding is in agreement with a number of previous studies [15, 16, 18, 23]. The finding that an elevation of blood pressure is a risk factor for the development of a cerebrovascular disease is well documented [13, 27–30].

### Strengths and limitations

This cohort of patients who were assessed by the EMS within the catchment area is large and representative. Since the data are based on a retrospective observational study, the results and the conclusion are dependent on the quality of the reporting. This quality most probably varies and is dependent on situational factors, as well as the experience and skills of the EMS staff. Information is missing for a large number of variables and this was adjusted for by multiple imputations.

Although the data are representative of the catchment area, they are collected from an EMS system within an urban area. For this reason, our results cannot be extrapolated to a national perspective where rural areas must be included as well.

The performance of the tools used to measure body temperature in the pre-hospital setting may be limited. Previous studies have suggested that these methods have a relatively high specificity but a limited sensitivity [31–33]. Furthermore, the environment in the pre-hospital setting is mostly not optimal for measurements of body temperature as compared with the intensive care unit, for example.

In this study, the categorisation of patients was linked to the RETTS triage system and the definition of the ESS code for dizziness. This definition did not enable the division of the symptoms of dizziness into subcategories, which may be regarded as a weakness of the system. For this reason, the subcategorisation that was made was based on a retrospective analysis of the free text based on the ED physician’s assessment. It may well be that some patients suffered from a combination of different symptoms including dizziness but where the dominant symptom was something else. These patients would not be categorised to the ESS code for dizziness.

## Conclusion

Among 1536 patients who were categorised by the EMS to the ESS code for dizziness, 6.2% had a time-sensitive aetiology. On the arrival of the EMS, eight factors were associated with the risk of having a time-sensitive aetiology. They were all linked to the type of symptoms or to clinical findings on the arrival of the EMS but also to clinical history. Further studies should aim to develop a risk-stratifying instrument in the pre-hospital setting and the validation of such an instrument.

## Supplementary Information

**Additional file 1: Table S1.** Multivariable analysis for complete cases (backward stepwise selection, *p* <  0.01 for staying in model). **Table S2.** Multivariable analysis in the subgroup Non-specific dizziness of variables included in the final model of the total group (except Rotary vertigo as type of dizziness).

## Data Availability

Data sets are available from the corresponding author on reasonable request.
