# Supplementary material for: Prediction of a time-sensitive condition among patients with dizziness assessed by the emergency medical services
Source: BMC Emerg Med. 2021 Mar 25;21:38. doi: 10.1186/s12873-021-00423-5 (PMC7995789; doi:10.1186/s12873-021-00423-5)
Supplement: Supplementary file 1 — Additional file 1: Table S1. Multivariable analysis for complete cases (backward stepwise selection, p < 0.01 for staying in model). Table S2. Multivariable analysis in the subgroup Non-specific dizziness of variables included in the final model of the total group (except Rotary vertigo as type of dizziness). [file 12873_2021_423_MOESM1_ESM.docx]

**Supplementary Table 1**

| **Appendix 1. Multivariable analysis for complete cases (backward stepwise selection, p<0.01 for staying in model)** | | |
| --- | --- | --- |
|  | Complete cases (n=73+910) |  |
|  | OR (95% CI) | *P* |
| Male gender | 2.06 (1.24,3.52) | 0.005 |
| Systolic blood pressure (per mmHg) | 1.015 (1.006,1.024) | 0.0009 |
| Body temperature (per degree Celsius) | ----------------------- | --------- |
| Rotary vertigo | 0.32 (0.17,0.59) | 0.0003 |
| Sudden onset | 0.33 (0.19,0.56) | <0.0001 |
| Nausea, vomiting | 2.08 (1.20,3.60) | 0.009 |
| Sudden onset headache | 11.64 (4.08,33.22) | <0.0001 |
| History of head trauma | ----------------------- | --------- |
| Treatment with anticoagulants | 2.71 (1.51,4.86) | 0.0008 |
| OR: odds ratio; CI: confidence interval | | |

**Supplementary Table 2**

| **Multivariable analysis in the subgroup Non-specific dizziness of variables** | | |
| --- | --- | --- |
| **included in the final model of the total group (except Rotary vertigo as type of dizziness)** | | |
|  | Multiple imputations (n=50+427) | |
|  | OR (95% CI) | p |
| Systolic blood pressure (per mmHg) | 1.016 (1.006,1.027) | 0.0024 |
| Body temperature (per degree Celsius) | 0.52 (0.31,0.87) | 0.0132 |
| Sudden onset | 0.31 (0.15,0.61) | 0.0008 |
| Nausea, vomiting | 1.64 (0.84,3.21) | 0.1481 |
| Sudden onset headache | 6.51 (1.70,24.91) | 0.0063 |
| History of head trauma | 3.02 (1.21,7.55) | 0.0177 |
| Treatment with antithrombotic agents or blood coagulation disorders | 2.80 (1.33,5.90) | 0.0067 |
|  |  |  |
